# Prolonged fasting-induced metabolic signatures in human skeletal muscle of lean and obese men

**DOI:** 10.1371/journal.pone.0200817

**Published:** 2018-09-05

**Authors:** Ann Mosegaard Bak, Mikkel Holm Vendelbo, Britt Christensen, Rikke Viggers, Bo Martin Bibby, Jørgen Rungby, Jens Otto Lunde Jørgensen, Niels Møller, Niels Jessen

**Affiliations:** 1 Medical Research Laboratory, Department of Clinical Medicine, Aarhus University, Aarhus, Denmark; 2 Department of Endocrinology and Internal Medicine (MEA), Aarhus University Hospital, Aarhus, Denmark; 3 Department of Nuclear Medicine and PET Center, Aarhus University Hospital, Aarhus, Denmark; 4 Research Laboratory for Biochemical Pathology, Department of Clinical Medicine, Aarhus University, Aarhus, Denmark; 5 Department of Public Health, Aarhus University, Aarhus, Denmark; 6 Department of Biomedicine, Aarhus University, Denmark; 7 Department of Clinical Pharmacology, Aarhus University Hospital, Denmark; Garvan Institute of Medical Research, AUSTRALIA

## Abstract

Insulin resistance is a well-known physiological adaptation to prolonged fasting in healthy skeletal muscle. Obesity is associated with insulin resistance and metabolic inflexibility in skeletal muscle, and a pronounced increase in the risk of metabolic complications. Under the hypothesis that the metabolic traits of insulin resistance associated with prolonged fasting are different from insulin resistance associated with obesity, we examined nine obese and nine lean participants during 12 and 72h of fasting, respectively. Insulin resistance in obese participants was associated with impaired insulin signaling, and reduced levels of glucose-6-phosphate and TCA-cycle intermediates. 72h of fasting in lean participants reduced insulin-stimulated glucose uptake to levels similar to obese participants fasted for 12h. This was associated with increased lipid oxidation, but not accumulation of diacylglycerol or acylcarnitines and impairment of insulin signaling. Prolonged fasting was associated with pronounced increases in β-hydroxybutyrate and β- hydroxybutyrylcarnitine levels in skeletal muscle suggesting augmented ketone body metabolism. Fasting induced insulin resistance may be a consequence of substrate competition. The underlying mechanism behind insulin resistance in obesity is thus not comparable to the physiological adaptations in skeletal muscle induced by prolonged fasting in lean participants.

## Introduction

Insulin sensitivity in skeletal muscle is closely coordinated by alterations in nutrient supply and activity levels, and this mechanism plays a central role in the regulation of whole body energy homeostasis [1]. During conditions of energy surplus and physical inactivity, skeletal muscle becomes insulin resistant; this condition may progress into type 2 diabetes and cardiovascular diseases.

Insulin resistance can be physiological response to a shift in substrate oxidation from a mixture of lipid and glucose to predominantly lipid intermediates [2]. This is classically observed in human skeletal muscle during prolonged fasting [3], and has been demonstrated in both lean [4,5], and obese participants [6,7]. There exists a strong association between elevated levels of FFA and insulin resistance in skeletal muscle [8–10]. In obesity, this correlates with reduced activation of the proximal insulin cascade [11]. Lipid infusions can elevate diacylglycerol (DAG) content in insulin resistant human skeletal muscle [12,13], and increased DAG content has been observed in insulin resistant participants [13]. In contrast, trained athletes have increased intramyocellular lipid content in association with increased insulin sensitivity [14,15]. Similarly, mice overexpressing diacylglycerol (DAG) acyltransferase 1 accumulate triacylglycerides (TG) and retain insulin sensitivity in combination with reduced DAG and ceramide levels [16]. This suggests that accumulation of TG in muscle can be a physiological condition, whereas accumulation of intermediate lipid metabolites, such as DAG, impair normal physiological function [17].

MR spectroscopy of human muscle during prolonged fasting show accumulation of triglycerides (TG) during 60 h of fasting [4,18], indicating that the delivery of free fatty acids (FFA) during fasting exceeds the demand for its oxidation. Interestingly, reductions in insulin signaling after an overnight fast in muscle from obese participants are normalized during a 48 h fast [6]. In addition, it has been shown that the lower content of mitochondrial proteins prior to fasting in obesity is not further reduced during prolonged fasting [19]. This could indicate that prolonged fasting changes the metabolic signature from a pathological pattern associated with type 2 diabetes to a pattern that resembles the physiological response to nutrient deprivation.

The objective of this study was to explore substrate metabolism in skeletal muscle from lean and obese participants during 12 and 72 h of fasting. The underlying hypothesis was that the metabolic traits of insulin resistance associated with prolonged fasting involves substrate competition, and not impaired insulin signaling.

## Subjects and methods

### Ethical approval

The study was approved by the Central Denmark Region Scientific Ethics Committee (M-2010-0182) and performed in compliance with the Declaration of Helsinki II. Written informed consent was obtained from all participants.

The study is registered in ClinicalTrials.gov with registration number NCT01299831. Not all the conditions from the originally registered trial are included in this manuscript.

### Study participants

18 apparently healthy young men aged from 20 to 35 years participated; nine lean (BMI 19–23) and nine obese (BMI 32–40 kg/m^2^). Before the study each subject completed a medical examination, including routine blood chemistry. All participants had low levels of physical activity, were non-smokers and used no prescription medications.

### Physical activity levels

Average duration and type of daily physical activity was registered by the participants on a questionnaire giving the following choices of physical activity: walking, running, biking (transportation), biking (exercise), ball game, gymnastics, fitness/weight training or other exercise. Walking and biking (transportation) was awarded 1 point per hour of physical activity (light activity) and the other types of activities (strenuous) were awarded 2 points per hour and the points were added, resulting in one total score for each individual.

### Study protocol

Data from this study regarding regulation of protein and lipid metabolism has previously been reported [20] and the design is summarized here: In a randomized crossover design, participants were examined on two occasions separated by a minimum of 21 days: 1) in the post absorptive state during an overnight fast of 12 h (control); 2) during 72 h of fasting (prolonged fasting).

During fasting participants were allowed to drink water and perform normal ambulatory activities. Daily blood samples (glucose, insulin, FFA) were taken to ensure compliance with the fast. On each study day, participants were studied during a four h non-insulin-stimulated period (*t* = 0–240), followed by a two h hyperinsulinemic euglycemic clamp (HEC) (*t* = 240–360) [21]. During a constant infusion of insulin (Actrapid, Novo Nordisk, Denmark) at the rate of 30 mU insulin/m^2^/min, plasma glucose levels were clamped at ~5 mM by adjusting the infusion rate of 20% glucose according to plasma glucose measurements every 10 min. Glucose infusion rate (GIR) during the last 30 min of HEC reflects peripheral insulin sensitivity and is expressed both per lean body mass (LBM) and total body weight (TBW) (kg).

### Skeletal muscle biopsies

At t = 60 and 270 min., under sterile conditions and using local anesthesia, a muscle specimen of approximately 100 mg was obtained from vastus lateralis of the quadriceps femoris muscle using a modified Bergström method [22]. The muscle tissue was immediately cleaned from blood, visible fat and connective tissue, snap frozen in liquid nitrogen, and stored at -80°C. Muscle tissue was subsequently freeze-dried and dissected free of fat and connective tissue under a microscope.

### Body composition

Body composition was determined by dual-energy X-ray absorptiometry scan (QDR-2000; Hologic, USA) [23].

### Blood analysis

HbA1c, ALAT and TSH were analyzed at Department of Clinical Biochemistry, Aarhus University Hospital. HbA1c was analyzed by HPLC, ALAT by absorption photometry and TSH by a luminometric assay.

Plasma glucose was measured immediately on an YSI 2300 STAT Plus glucose analyzer (YSI®). Insulin, C-peptide, and cortisol were measured by ELISA (DAKO, Agilent Technologies, Denmark (insulin), ALPCO, NH, USA (C-peptide) and DRG, NJ, USA (cortisol)). Glucagon was analyzed by RIA (EMD Millipore, Germany). Commercial kits were used to analyze FFA (Wako Chemicals, Germany), glycerol and β-hydroxybutyrate (Cayman Chemicals, USA). Adiponectin and leptin were analysed by validated in-house time-resolved immunofluorometric assays based on commercial reagents (R&D Systems, UK) [24].

### Indirect calorimetry

The respiratory exchange ratio (RER) and resting energy expenditure (EE) were measured by indirect calorimetry (Deltatrac monitor, Dantes Instrumentarium, Finland) performed at *t* = 180–210 and *t* = 300–330. Mean values of the last 25 min were used for calculations. Urine was collected during the entire study day and urea content was measured [25]. Glucose and lipid oxidation were estimated after correction for protein oxidation, calculated based on urea nitrogen excretion [26].

### Glucose metabolism

A primed infusion of [3-^3^H]-glucose (bolus 20 μCi, 0.20 μCi/min; Perkin Elmer, Belgium) was initiated at *t* = 0 min and continued throughout the study day.

Glucose rates of appearance (R_a_) and disappearance (R_d_) were calculated at 15 min. intervals from *t* = 210–240 and *t* = 330–360 min. using Steele’s non-steady state equations [27].

Although glucose is distributed in all tissue fluids, the model assumes that the tracer is distributed in a single compartment pool. Additionally it is assumed that the tracer is readily and uniformly mixed with the endogenous pool of tracee, that metabolism of tracer and trace are identical, that concentrations and rate of appearance of tracer and trace are sampled from the same region, that Ra_glu_ is slower than mixing time in the compartment, and that time and concentration changes are restricted to changes in clearance [28].

Ra_glu_ was calculated as:

Raglu=([3−3H]−glucoseinfusionrate/SA)–((Vd*c)/SA*(ΔSA/Δt))

in which SA is the specific activity for [3-^3^H]-glucose, the total concentration of glucose, Δt the time between samplings and Vd the total volume of distribution. Vd was assumed to remain constant and calculated as [28]:

Vd=weight(kg)*p*Vr

in which a pool fraction (p) of 0.65 and volume of distribution for glucose (Vr) of 200 ml/kg was used. Then glucose rate of disappearance can be calculated as:

Rdglu=Raglu–Vd*(Δc/Δt)

Endogenous glucose production (EGP) equals R_a_ under non-insulin-stimulated conditions, and was calculated by subtracting GIR from R_a_ during the HEC.

Non-oxidative glucose disposal (NOGD) was calculated as R_d_ minus oxidative glucose disposal. Muscle glycogen content was determined as previously published [29].

### Western blot analysis

Western Blotting was performed on freeze dried muscle tissue as previously described [20] using Stain-Free technology as control for equal loading [30] and an internal standard to control for blot to blot variability.

Antibodies (numbers in parentheses indicate catalog numbers) against panAKT (4691), AKT p-Ser^473^ (9271), AKT p-Thr^308^ (9275), Akt substrate of 160 kDa (AS160) p-Thr^642^ (4288), Glycogen Synthase (GS) (3886), GS p-Ser^641^ (3891), GS kinase (GSK)3 α/β (5676), GSK3 α/β p-Ser^21/9^ (9331), AMPK p-Thr^172^ (2531) and HKII (2867) were from Cell Signaling Technology (CA, USA). Antibodies against panAMPK (07–181), AS160 (07–741), and Acetyl-CoA carboxylase (ACC) p-Ser^79^ (07–303) were from Merck Millipore. ACC protein was detected using HRP-linked streptavidin (R&D Systems, USA).

### Metabolomics

Non-targeted Metabolomic profiling was performed by Metabolon® as previously described [31]. Non-insulin-stimulated muscle samples from both examination days were analyzed using a non-targeted gas chromatography–mass spectrometry and liquid chromatography–mass spectrometry based metabolomic quantification protocol.

### Statistical analysis

Normal distribution of baseline data was assessed by inspection of QQ-plots. Data are presented as means ± SD or median (range) for non-normally distributed data. Baseline characteristics were analyzed using student’s t-test, or in case of skewed distribution Wilcoxon Rank sum test. The effects of BMI (lean vs. obese), fasting (12 h vs. 72 h fast), and insulin (non-insulin-stimulated vs. HEC) and their interactions on dependent variables were assessed using a mixed-effect three-way ANOVA with repeated measures or with two-way ANOVA with repeated measures, identical to the three-way model except without the effect of time when relevant. The within-subject design (repeated measures on the same subject within and between study days) was accounted for in the model by including subject ID, subject ID x intervention and subject ID x time as random effects. Pairwise comparison of means with no adjustment for multiple comparisons was performed post-hoc using the command ‘pwcompare’ in STATA to compare differences within and between individual conditions. Normal distribution and adequacy of the mixed model to describe data was assessed by inspection of QQ plots of residuals. The level of significance was set at P<0.05. Statistical analyses were performed using Stata (Stata 13.1, StataCorp, USA). Graphs were designed in SigmaPlot (SigmaPlot 11.0, USA).

Statistical analysis of the metabolomics data was carried out by Metabolon® by two-way ANOVA following normalization to tissue weight, median scaling, imputation of missing values with the minimum observed value for each compound, and log transformation of median scaled data. False discovery rate was estimated using *q* values to account for multiple comparisons.

## Results

All underlying data relevant to this paper has been uploaded to Dryad, DOI:10.5061/dryad.6121hj7. Anthropometric data of the participants have previously been published [20] and are summarized in Table 1. HbA1c was within the normal range for all participants (25–36), although mean HbA1c in mmol/mol was ~10% higher in obese vs. lean participants.

**Table 1:** Anthropometric data.

	Lean (n = 9)	Obese (n = 9)	P-value
**Age (years)**	24 (21–33)	24 (21–35)	1.0
**Weight (kg)**	69.2±5.3	122.9±12.6	<0.001
**BMI (kg/m**^**2**^**)**	21.4±1.1	35.7±2.6	<0.001
**LBM (kg)**	57.3±3.7	78.5±6.5	<0.001
**Weight loss during 72 h of fasting (kg)**	3.7±1.7	4.4±2.3	0.529
**Surface area (m**^**2**^**)**	1.87±0.09	2.45±0.2	<0.001
**Fat mass (kg)**	10.2 (7.4–14.0)	41.1 (34.1–54.7)	<0.001
**HbA1c (mmol/mol)**	30 (25–35)	33 (30–36)	0.027
**HbA1c (%)**	4.9 (4.4–5.4)	5.2 (4.9–5.4)	
**ALAT (U/l)**	31±17	45±19	0.105
**TSH (10**^**3**^ **IU/l)**	2.4 (1.6–3.7)	2.0 (1.3–3.8)	0.426
**Physical activity level (points)**	7.9±3.4	8.9±4.1	0.58

Normally distributed data were analyzed using student’s t-test and presented as mean ± SD. Non- normally distributed data were analyzed using Wilcoxon Rank sum test and presented as a median (range).

*P<0.05 compared to lean.

Some of these data have previously been published [20].

### Hormones and metabolites

Plasma levels of hormones and metabolites are presented in Table 2. Non-insulin-stimulated insulin levels, were ~2–3 fold higher in obese than lean and decreased by at least 50% during 72 h of fasting in both groups. During the HEC, insulin levels were ~15–30% higher in obese than lean.

**Table 2:** Plasma levels of hormones and metabolites.

	LEAN				OBESE				P-values ANOVA
	12 h fast		72 h fast		12 h fast		72 h fast		
	- insulin	+ insulin	- insulin	+ insulin	- insulin	+ insulin	- insulin	+ insulin	
Insulin (pM)	23 (10–64)	234* (188–319)	11^#^ (10–20)	257* (116–293)	64^+^ (27–105)	305*^+^ (218–391)	20^#^ ^+^ (10–95)	299* (235–915)	BMI x insulin: P<0.01 Fast x insulin: P<0.01
C-peptide (pM)	350 ± 131	298 ± 152*	212 ± 279^#^	330 ± 288*	499 ± 157	494 ± 103^+^	206 ± 88^#^	523 ± 186*^+^	BMI x fast x insulin: P<0.05
FFA (mM)	0.39 ± 0.17	0.05 ± 0.03*	1.47 ± 0.26^#^	0.19 ± 0.09*^#^	0.55 ± 0.09^+^	0.11 ± 0.03*	1.07 ± 0.14^#^ ^+^	0.31 ± 0.09*^#^	BMI x fast x insulin: P<0.01
Glucose (mM)	4.9 ± 0.3	5.0 ± 0.3*	3.2 ± 0.2^#^	5.1 ± 0.3*	4.8 ± 0.4	5.2 ± 0.3	3.6 ± 0.4^#^ ^+^	5.3 ± 0.3*^#^ ^+^	BMI x fast x insulin: P<0.01
Glucagon (ng/L)	61.3 ± 28.6	51.9 ± 23.8*	144.7 ± 35.3^#^	85.6 ± 33.3*^#^	86.1 ± 27.8	79.8 ± 24.9^+^	136.4 ± 46.2^#^	109.0 ± 42.9*^#^	BMI x fast x insulin: P<0.05
β-hydroxy- butyrate (mM)	0.3 ± 0.1		3.0 ± 0.6^#^		0.3 ± 0.2		1.9 ± 0.6^#^		BMI x fast: P<0.05
Glycerol (mg/L)	5.1 ± 1.0		10.7 ± 5.6^#^		6.2 ± 1.3^+^		6.8 ± 2.2^+^		BMI x fast: P<0.01
Adiponectin (mg/L)	13.1 ± 14.1		12.0 ± 3.5^#^		8.0 ± 1.8^+^		7.3 ± 1.7^+^		BMI: P<0.001 Fast: P<05
Leptin (μg/L)	2.5 (1.0–5.2)		1.0^#^ (0.8–2.9)		27.9^+^ (7.4–62.0)		9.9^+^^#^ (2.6–17.5)		BMI: P<0.001 Fast: P<0.001

Normally distributed data are presented as mean ± SD. Non-normally distributed data are presented as median (range). Insulin and glucagon were determined at *t* = 60 (non-insulin-stimulated period) and *t* = 270 (HEC) after 12 and 72 h of fasting in lean and obese participants. FFA and glucose were measured in triplicate and C-peptide in duplicate during that last 30 min of the non-insulin-stimulated and HEC period. Ketones (β-hydroxybutyrate), glycerol, adiponectin and leptin were measured at *t* = 0.—insulin = non-insulin-stimulated conditions

+ insulin = hyperinsulinemic euglycemic clamp conditions (HEC). Post hoc test

*P<0.05 compared to non-insulin-stimulated conditions

^+^P<0.05 compared to lean

^#^P<0.05 compared to 12 h fast.

Prolonged fasting prompted a greater decrease in non-insulin-stimulated plasma glucose levels in lean (~35%) than obese (~25%,) despite higher insulin levels in obese. As per protocol, the glucose levels during HEC were close to five mmol/l on all study days. However, post-hoc tests revealed slightly, but statistically significantly, higher levels in obese during 72 h of fasting compared to both 12 h fast within obese and lean during 72 h fast.

Insulin-stimulation suppressed FFA levels significantly in both groups, but less so during prolonged fasting, resulting in ~3–4 fold higher plasma FFA levels during the HEC during 72 h of fasting compared to 12 h of fasting. Non-insulin-stimulated plasma FFA concentrations have previously been published [20], and were higher in obese during an overnight fast, but lower during prolonged fasting.

During prolonged fasting the ketone body, β-hydroxybutyrat (β-OHB), was highly elevated in plasma and more so in lean than obese. Plasma glycerol levels were higher in obese during 12 h of fasting, and increased ~50% more in lean than obese during prolonged fasting.

To assess a potential hormonal cross-talk between adipose tissue and skeletal muscle, we determined circulating levels of the adipokines adiponectin and leptin. Adiponectin was higher in lean than obese and decreased during prolonged fasting. Leptin was lower in lean and decreased during prolonged fasting.

### Glucose kinetics and insulin sensitivity

Glucose tracer data are presented in Table 3. The following describes the data presented per lean body mass. Whole body insulin sensitivity, determined by HEC, was ~50% lower in obese than lean during 12 h of fasting. 72 h of fasting reduced GIR to similar absolute levels in the two groups reflecting a much larger decrease in insulin sensitivity in lean (~60%) than obese (~30%) and a BMI x fasting interaction. Similarly, insulin-stimulated R_d_, reflecting insulin sensitivity in peripheral tissues, was ~45% lower in obese than lean during 12 h of fasting, and prolonged fasting decreased insulin-stimulated R_d_ ~50% in lean as opposed to ~25% in obese. Non-insulin-stimulated R_d_ was ~22% lower in obese than lean during 12 h of fasting. During prolonged fasting R_d_ was reduced in both groups with no persisting differences between lean and obese.

**Table 3:** Glucose tracer data.

	LEAN				OBESE				P-values ANOVA
	12 h fast		72 h fast		12 h fast		72 h fast		
	- insulin	+ insulin	- insulin	+ insulin	- insulin	+ insulin	- insulin	+ insulin	
GIR (mg/kg LBM/min)		6.0 ± 1.9		2.4 ± 0.8^#^		3.0 ± 0.9^+^		2.0 ± 0.6^#^	BMI x fast: P<0.05
GIR (mg/kg TBW/min)		5.1 ± 2.5		2.1 ± 0.7^#^		1.9 ± 0.6^+^		1.3 ± 0.4^#^	BMI x fast: P<0.01
R_d_ glucose (mg/kg LBM/min)	2.3 (2.1–2.5)	6.4* (5.9–8.1)	1.4^#^ (1.3–1.4)	3.0*^#^ (2.7–3.4)	1.8^+^ (1.7–1.9)	3.6*^+^ (3.1–4.1)	1.4^#^ (1.3–1.4)	2.7*^#^^+^ (2.3–2.8)	BMI x fast: P<0.05 Insulin x BMI: P<0.01 Insulin x fast: P<0.05
R_d_ glucose (mg/kg TBW/min)	1.9 (1.4–2.5)	5.8* (3.0–11.5)	1.2^#^ (1.1–1.3)	2,7*^#^ (1.9–3.8)	1.1^+^ (0.8–1.2)	2.3*^+^ (1.3–2.9)	0.9^#^^+^ (0.7–1.0)	2.0*^#^^+^ (1.6–3.2)	BMI x fast: P<0.05 Insulin: P<0.01
EGP (mg/kg LBM/min)	2.29 ± 0.42	0.35* ± 0.35	1.36^#^ ± 0.15	0.60 ± 0.22*	1.74^+^ ± 0.27	0.53* ± 0.36	1.33^#^ ± 0.18	0.63* ± 0.22	BMI x fast x insulin: P<0.01
EGP (mg/kg TBW/min)	1.89 ± 0.36	0.22* ± 0.20	1.22^#^ ± 0.10	0.53* ± 0.20	1.05^+^ ± 0.17	0.31* ± 0.23	0.85^#^^+^ ± 0.18	0.40*^#^ ± 0.24	BMI x fast x insulin: P<0.01

Normally distributed data are presented as mean ± SD. Non-normally distributed data are presented as median (range). Insulin sensitivity (GIR) was assessed during the last 30 min. of the HEC. Insulin was infused at a rate of 30 mU/m^2^/min. Rd of glucose and EGP were measured by the use of glucose tracer techniques during steady state in that last 30 min of the non-insulin-stimulated and HEC period.

- insulin = non-insulin-stimulated conditions

+ insulin = hyperinsulinemic euglycemic clamp conditions (HEC).

Post hoc test

*P<0.05 compared to non-insulin-stimulated conditions

^+^P<0.05 compared to lean

^#^P<0.05 compared to 12 h fast.

When the data are presented per TBW the patterns are similar but the means are lower in the obese relative to the lean participants compared to when presented by LBM.

EGP, measured as R_a_ of glucose, was significantly lower in obese than lean during 12 h of fasting. Prolonged fasting reduced EGP in both groups, although the decrease was more pronounced in lean (~40%) than obese (~25%). EGP was suppressed to equal levels by insulin in both groups during 12 and 72 h of fasting, demonstrating preserved hepatic insulin sensitivity.

### Substrate oxidation rates

Resting energy expenditure (EE) was significantly lower in obese participants, and non-insulin-stimulated EE increased slightly during prolonged fasting in both groups (Table 4). During 12 h of fasting, respiratory exchange ratio (RER) increased during HEC in lean participants, demonstrating metabolic flexibility in lean only. RER decreased during prolonged fasting, and tended to be lower in obese than lean (BMI P = 0.069), reflecting a shift toward lipid oxidation.

**Table 4:** Indirect calorimetry.

	LEAN				OBESE				P-values ANOVA
	12 h fast		72 h fast		12 h fast		72 h fast		
	- insulin	+ insulin	- insulin	+ insulin	- insulin	+ insulin	- insulin	+ insulin	
EE/LBM (kcal/day/kg LBM)	30.1 ± 2.4	29.8 ± 1.8	31.3 ± 2.4^#^	29.6 ± 1.8*	28.5 ± 2.2^+^	27.6 ± 0.8^+^	30.2 ± 1.6^#^	28.4 ± 1.6*	Fast x insulin: P<0.5 BMI: P<0.05
RER	0.82 ± 0.04	0.85 ± 0.04*	0.74 ± 0.02^#^	0.73 ± 0.01^#^	0.80 ± 0.03	0.81 ± 0.03^+^	0.74 ± 0.01^#^	0.73 ± 0.02*^#^	Fast: P<0.01
Glucose Oxidation (mg/kg LBM/min)	1.55 ± 0.88	2.09 ± 0.93*	0.36 ± 0.46^#^	0.04 ± 0.21^#^	1.23 ± 0.41	1.35 ± 0.53^+^	0.34 ± 0.18^#^	0.05 ± 0.27*^#^	Fast x insulin: P<0.01 BMI x insulin: P = 0.069
Lipid Oxidation (mg/kg LBM/min)	0.97 ± 0.30	0.66 ± 0.29*	1.63 ± 0.19^#^	1.47 ± 0.10*^#^	1.04 ± 0.24	0.98 ± 0.26^+^	1.57 ± 0.15^#^	1.45 ± 0.12*^#^	Fast: P<0.01 Insulin: P<0.01

Normally distributed data are presented as mean ± SD. Non-normally distributed data are presented as median (range). Energy expenditure, Respiratory Exchange Ratio (RER), glucose oxidation and lipid oxidation were estimated by indirect calorimetry at *t* = 180–210 and *t* = 300–330.

- insulin = non-insulin-stimulated conditions

+ insulin = hyperinsulinemic euglycemic clamp conditions (HEC).

Post hoc test

*P<0.05 compared to non-insulin-stimulated conditions

^+^P<0.05 compared to lean

^#^P<0.05 compared to 12 h fast.

Prolonged fasting modified the effect of insulin on glucose oxidation (fasting x insulin interaction). Post-hoc test revealed that glucose oxidation during 12 h of fasting during HEC increased in lean only, and insulin failed to increase glucose oxidation in both obese and lean during prolonged fasting. Lipid oxidation increased during prolonged fasting and decreased during HEC. The decrease during insulin infusion was more pronounced in lean participants, but there was no statistically significant interaction.

### Non-targeted metabolomics

201 distinct metabolites involved in glucose and lipid metabolism were identified in skeletal muscle tissue. The metabolite data are listed in Table 5 of the manuscript and in the supplemental material (S1 Table).

**Table 5:** Myocellular metabolomics.

	Fold of change				Two-way ANOVA with Repeated Measures		
	ANOVA Contrasts						
Biochemical name	Fasted Control		Obese Lean		Fasting Main Effect	BMI Main Effect	Fasting BMI Inter-action
	Lean	Obese	Control	Fasted			
Glucose	0.73	0.8	0.92	1.00			
Glucose-6-phosphate	1.39	1.00	0.66	0.47			
Fructose-6-phosphate	1.38	1.07	0.62	0.48			
Pyruvate	0.85	1.01	0.80	0.94			
Lactate	0.96	1.2	0.66	0.81			
Maltotriose	0.67	1.13	0.54	0.90			
Citrate	0.99	1.10	0.71	0.79			
Malate	1.08	0.97	0.69	0.62			
Succinate	0.86	1.01	0.53	0.63			
Pyrophosphate (PPi)	0.96	0.92	0.99	0.94			
Phosphate	0.91	1.01	0.90	0.99			
NAD+	0.91	1.00	0.91	1.00			
NADH	1.06	1.36	0.70	0.90			
NADP+	1.17	1.27	0.93	1.01			
ATP	0.96	1.13	0.86	1.02			
ADP	1.04	0.99	0.87	0.83			
AMP	1.02	1.03	0.91	0.92			
Palmitate (16:0)	0.97	1.38	0.76	1.07			
Stearate (18:0)	1.02	1.16	0.75	0.86			
Oleate (18:1n9)	1.40	1.52	0.96	1.04			
Arachidate (20:0)	0.98	1.05	0.79	0.85			
Glycerol	1.25	1.21	0.88	0.86			
1,2-dipalmitoylglycerol	0.99	0.92	0.98	0.91			
1,3-dipalmitoylglycerol	1.01	1.30	0.92	1.19			
Carnitine	0.75	0.95	0.76	0.97			
Palmitoylcarnitine	0.80	0.60	1.50	1.12			
Acetylcarnitine	1.14	1.92	0.55	0.93			
3-hydroxybutyrate	19.66	19.27	0.74	0.73			
Hydroxybutyrylcarnitine	15.53	15.63	0.63	0.64			

Metabolite concentrations were determined in skeletal muscle tissue during non-insulin-stimulated conditions in lean and obese after 12 h (control condition) and 72 h of fasting (fasted condition). The data are presented as fold of change. The muscle biopsies were obtained at t = 60 min. Green indicates significant difference (*p*≤0.05) between the mean values of the groups compared; metabolite ratio of < 1.00. Light green indicates narrowly missed statistical cutoff for significance 0.05<p<0.10; metabolite ratio of < 1.00. Red indicates significant difference (*p*≤0.05) between the groups compared; metabolite ratio of ≥ 1.00. Light Red indicates narrowly missed statistical cutoff for significance 0.05<p<0.10: metabolite ratio of ≥ 1.00. Non-colored cell indicates that mean values are not significantly different for that comparison. Blue indicates significant (*p*≤0.05) ANOVA effect.

#### Metabolites of carbohydrate metabolism

Glucose levels in muscle tissue fell by ~25% during 72 h of fasting in both groups. Glucose 6-phosphate (G6P) and fructose-6-phosphate (F6P), the first two metabolites in the glycolytic pathway, were ~35% lower in obese than lean during 12 h of fasting. This difference was augmented during 72 h of fasting because of an increase in G6P and F6P levels under prolonged fasting in lean only. Further downstream in the glycolytic pathway, lactate levels were lower in obese participants while pyruvate levels, although slightly lower (~20%) in obese participants during 12 h fast, were not significantly affected by fasting or BMI.

Maltotriose, a product of glycogenolysis [32], was ~45% lower in obese during 12 h of fasting and was reduced in lean only with prolonged fasting (fasting x BMI interaction), resulting in similar absolute levels between lean and obese participants during prolonged fasting.

#### Mitochondrial metabolites

The TCA cycle intermediates citrate, succinate, and malate, were 20–45% lower in obese than lean individuals with no effect of duration of fasting. However, pyrophosphate and phosphate were not affected by BMI or fasting in skeletal muscle. Neither were the redox coenzymes of oxidative phosphorylation NAD+, NADH and NADP+ or the markers of energy levels, adenosine 5’triphosphate (ATP), adenosine 5’diphosphate (ADP), and adenosine 5’monophosphate (AMP).

#### Metabolites of lipid metabolism in skeletal muscle

Accumulation of several long chain FFAs in skeletal muscle was observed during the extended fast in both groups. However, FFA levels were not higher in skeletal muscle of obese compared to lean during both 12 and 72 h of fasting. In fact, palmitate, stearate and arachidate levels were lower during 12 h of fasting in obese, but levels were similar between groups during 72 h of fasting. Even though plasma-glycerol increased more during 72 h of fasting in lean than obese (Table 2), intramuscular glycerol levels increased similarly in lean and obese during 72 h of fasting. Interestingly, the diacylglycerols (DAGs) 1,2-dipalmitoylglycerol and 1,3-dipalmitoylglycerol were not elevated in muscle from obese participants compared to lean, nor did they increase during prolonged fasting.

Free carnitine, which binds to and assists the transportation of FFA into the mitochondria, was lower in obese than lean; this was most pronounced during 12 h of fasting (~25%). These levels reduced during prolonged fasting in lean only, resulting in similar levels between lean and obese during prolonged fasting. Long chain fatty acyl carnitines were unaffected by BMI and fasting. Acetylcarnitine, which transports surplus acetyl groups out of the mitochondria, was lower in obese than lean during 12 h of fasting, but increased to levels similar to lean during 72 h of fasting (fasting x BMI interaction).

In skeletal muscle, β-OHB and its derivative hydroxybutyrylcarnitine (β-OHB-carnitine) were elevated ~15–20 fold in both lean and obese participants during prolonged fasting compared to 12 h of fasting, indicating a high availability of ketones as substrate for metabolism in muscle tissue.

### Glycogen concentrations and metabolism

**Fig 1:**
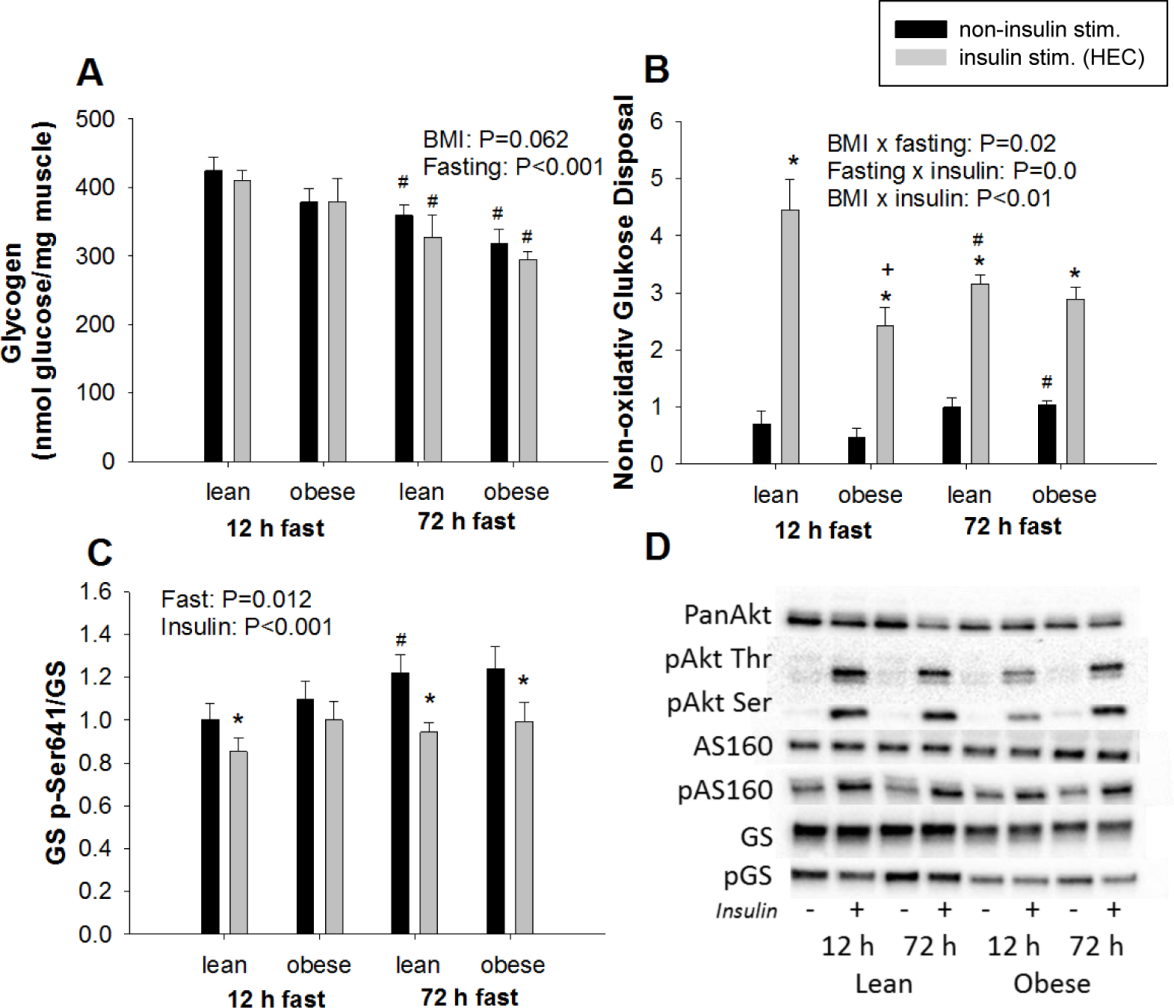
Glycogen content and regulators/markers of metabolism determined after 12 and 72 h of fasting. Data are presented as mean±SD. **A**: Glycogen levels were significantly reduced by 72 h of fasting (fasting effect P<0.001) and tended to be lower in the obese group than the lean (BMI effect P = 0.062). **B**: There were three 2-way interactions between the effects of BMI, fasting and insulin on rate of whole body glycogen synthesis measured as non-oxidative glucose disposal (NOGD). NOGD significantly increased by insulin (P<0.001). **C**: 72 h of fasting increased phosphorylation of GS p-Ser^641^ reflecting decreased activity of the enzyme (fasting effect P = 0.012), whereas insulin increased GS activity (insulin effect P<0.001). **D**: Representative Western blots. *P<0.05 compared to non-insulin-stimulated conditions, ^+^P<0.05 compared to lean, ^#^P<0.05 compared to 12 h fast.

Glycogen levels in skeletal muscle were reduced by ~20% during 72 h of fasting and tended to be lower in obese than lean (P = 0.062) (Fig 1). The rate of whole body glycogen synthesis, measured as NOGD, was significantly increased by insulin; however, fasting-induced changes in insulin response differed between lean and obese. Insulin-stimulated NOGD was ~45% lower in obese than lean during 12 h of fasting and decreased in response to prolonged fasting in lean only. Non-insulin-stimulated NOGD increased in both groups during prolonged fasting, but this increase only reached statistical significance in obese participants. GS is the rate limiting enzyme in glycogenesis, and the activity of GS is inhibited by phosphorylation on Ser^641^ [33]. 72 h of fasting increased phosphorylation of pGS Ser^641^, and insulin reduced GS phosphorylation independently of BMI and duration of the fast.

### Insulin signaling to glucose uptake in skeletal muscle

**Fig 2:**
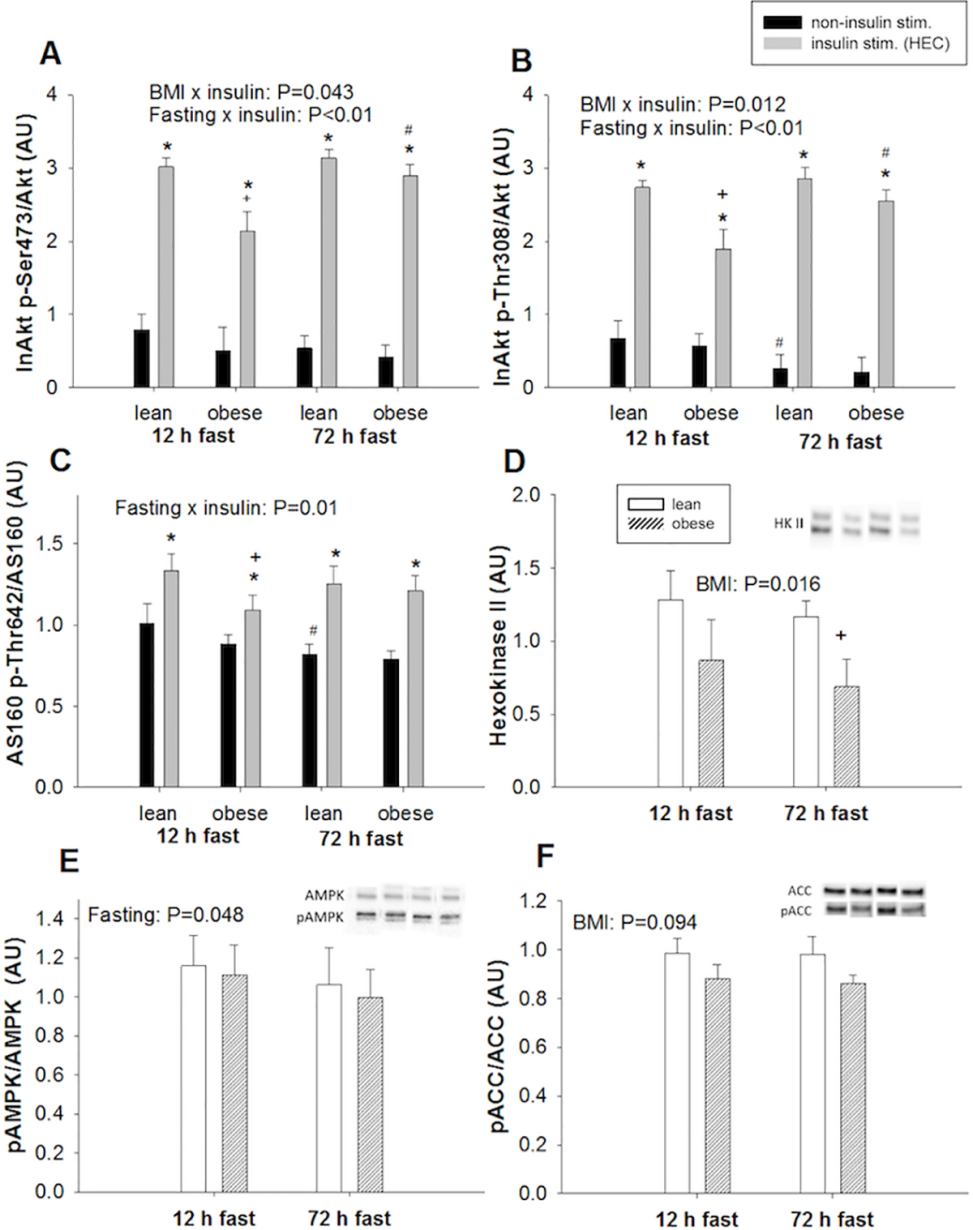
Protein levels and phosphorylations of Akt, AS160, hexokinase, AMPK and ACC. Phosphorylation of Akt and AS160 were assessed by Western blots in muscle biopsies taken before (black bars) and during (grey bars) insulin stimulation after 12 and 72 h of fasting in lean and obese participants. Protein levels of Hexokinase II, as well as phosphorylations of AMPK and ACC were assessed in non-insulin-stimulated muscle biopsies from lean (white bars) and obese participants (crossed bars) after 12 and 72 h of fasting. Data are presented as mean±SD. **A** and **B**: Insulin stimulation significantly increased phosphorylation on both pAKt Ser^473^ and Thr^308^ regardless of BMI and duration of the fast. We found significant BMI x insulin and fasting x insulin interactions on both phosphorylation sites. Post hoc tests revealed that insulin-stimulated Akt phospylation was lower in obese than lean after 12 h of fasting (P<0.01) but increased during 72 h of fasting. In lean, Akt phosphorylation levels were not affected by fasting. **C**: We found an interaction between the effect of insulin and fasting (fasting x insulin P = 0.01) on pAS160 Thr^642^/AS160, and post hoc test revealed a lower insulin-stimulated phosphorylation level in obese after 12 h of fasting compared to lean (P = 0.047). Similar to Akt, the insulin-stimulated levels in obese increased during 72 h of fasting to levels comparable to lean. **D**: Protein levels of Hexokinase II (relative to total amount of protein) were lower in obese than lean (P = 0.016). **E:** pAMPK Thr^172^/AMPK decreased slightly during 72 h of fasting with no difference between groups (fasting effect P = 0.048) **F**: pACC/ACC was not significantly affected by BMI or fasting. Representative blots in panel D, E and F have been cut to remove insulin-stimulated biopsies. *P<0.05 compared to non-insulin-stimulated conditions, ^+^ P<0.05 compared to lean, ^#^ P<0.05 compared to 12 h fast.

Insulin-stimulated glucose uptake in skeletal muscle through activation of an intracellular signaling cascade ultimately leading to translocation of GLUT4 vesicles to the sarcolemma [34]. Insulin stimulation significantly increased phosphorylation on the two activating sites on Akt: Ser^473^ and Thr^308^, but the effect of insulin differed among BMI groups and fasting states. Insulin-stimulated phosphorylation levels were significantly lower in obese than lean participants during 12 h of fasting, but the effect of insulin in obese was normalized during 72 h of fasting (Fig 2). In contrast, the insulin-stimulated phosphorylation levels in lean were not affected by prolonged fasting.

Next, we assessed phosphorylation of Thr^642^ on AS160, a direct target of Akt that regulates GLUT4 translocation, and found that insulin stimulation significantly increased phosphorylation at this site, also. Similar to Akt, insulin-stimulated phosphorylation levels were lower in obese than lean during 12 h of fasting but similar between groups during prolonged fasting.

Protein levels of the hexokinase II, which catalyzes the conversion of glucose to glucose 6-phosphate, was significantly lower in obese than lean (Fig 2).

### AMPK and ACC phosphorylation in skeletal muscle

Phosphorylation levels on the energy sensor AMPK at Thr^172^ in the non-insulin-stimulated state decreased marginally during prolonged fasting, indicating that skeletal muscle energetics were preserved (Fig 2). We also examined phosphorylation of ACC, a bona fide target of AMPK, and found no significant effect of prolonged fasting.

## Discussion

This study illustrates the metabolic shifts in human skeletal muscle associated with apparent contrasting forms of insulin resistance: the acute, physiological response to prolonged fasting and the chronic form associated with obesity. Although the magnitude of insulin resistance induced by prolonged fasting in lean participants was similar to overnight fasted participants with modest obesity, the metabolic signatures and insulin signaling data reveal distinct differences. Therefore, our data challenge some of the existing theories of mechanisms for insulin resistance in skeletal muscle.

As demonstrated in this study and by others [35,36], reduced insulin-stimulated glucose uptake in skeletal muscle of obese participants during 12 h of fasting is associated with increased lipid oxidation. This has been proposed in ‘the glucose fatty-acid cycle’ hypothesis by Randle et al in 1963 as the underlying mechanism for reduced insulin-stimulated glucose uptake [37]. According to Randle the increased lipid oxidation would lead to increased mitochondrial NADH/NAD^+^ ratio and citrate concentrations and increased G6P levels, but we found no evidence of such increases. In fact, in the insulin resistant state of obesity, we found lower levels of citrate and G6P, and the NADH/NAD^+^ ratio was not affected by BMI. In addition, we found that insulin resistance induced in lean individuals by prolonged fasting led to an increase in G6P content without concomitant increases in citrate. Thus, our data do not support that the proposed mechanisms in ‘the glucose fatty-acid cycle’ hypothesis cause reduced insulin-stimulated glucose uptake during prolonged fasting or insulin resistance associated with obesity.

Glucose phosphorylation, catalyzed by hexokinase, is the first committed step in glucose uptake in skeletal muscle. Interestingly, protein levels of hexokinase II were lower in obese compared to lean, and the lower capacity to phosphorylate glucose after entry into the muscle may contribute to the reduced insulin sensitivity. This is in agreement with previous measures of reduced hexokinase mRNA levels and activity in obese and type 2 diabetic participants [38,39]. Reduced levels of G6P in insulin resistant muscles have also been measured using NMR-spectroscopy [40]. This has formed the basis of a theory where increases in plasma FFA concentrations lead to accumulation of DAGs, which inhibit proximal insulin signaling [41,42]. We did observe a modest decrease in insulin-stimulated Akt phosphorylation in the obese participants during an overnight fast, indicating reduced proximal insulin signaling. However, prolonged fasting increased insulin-stimulated Akt phosphorylation in obese despite augmented insulin resistance. This paradoxical increase in insulin-stimulated Akt phosphorylation during prolonged fasting has previously been observed in obese participants [6] and contradicts a role of proximal insulin signaling in insulin resistance associated with prolonged fasting. This is further supported by our present and previous Akt phosphorylation data from lean participants [4,6]. Previous studies have suggested that high fasting insulin levels drive insulin resistance in obesity [43]. The improved insulin-stimulated Akt phosphorylation in the obese after prolonged fasting could be related to the reduction insulin levels. However, these changes do not translate into increased insulin sensitivity (GIR) in the obese after prolonged fasting.

Unlike previous reports, the present investigations are supported by measurements of intramyocellular metabolites. We found no elevation of two species of DAG in the insulin resistant obese group, nor did the levels of the same DAG species increase with insulin resistance induced by prolonged fasting in the lean group. Thus, our data do not give evidence to the theory that elevated levels of DAGs play a mechanistic role in the development of insulin resistance in skeletal muscle in obesity or during prolonged fasting. However, we did observe reduced insulin signaling in obese during 12 h of fasting and this resembles insulin resistance in T2D participants [44,45]. Our data indicate that several forms of insulin resistance exist and caution should be taken in the pursuit of unifying underlying mechanisms.

The metabolism of long-chain fatty acids plays a central role in the mechanistic theories behind insulin resistance in muscles. In this study, however, despite our previous observations of a higher lipolytic rate during 12 h of fasting in obese participants [20], we did not find increased FFA content in skeletal muscle from obese participants at any time point. The increased fat mass in obese was associated with higher levels of leptin and lower levels of adiponectin which is in agreement with the observed insulin resistance. Prolonged fasting reduced circulating leptin levels although at a higher level in the obese participants. Leptin can negatively regulate lipolysis [46]. The higher leptin levels may therefore be involved in the reduced increase in lipolytic rate in obese compared to lean during prolonged fasting [20]. In spite of an abundant supply of long-chain fatty acids from plasma during prolonged fasting, we found only modest increases in long chain fatty acids and no increases in acylcarnitines in skeletal muscle. We have previously shown that prolonged fasting increases triglyceride accumulation in skeletal muscle [4], and we therefore speculate that a substantial amount of the long-chain fatty acids are reesterified and not oxidized. The increased lipid oxidation during prolonged fasting can be at least partly due to oxidation of ketone bodies instead of long-chain fatty acids and this could provide an alternative explanation for a mechanism behind fasting induced insulin resistance. Animal studies have shown direct inhibitory effects of ketone bodies on glucose uptake, phosphorylation, and glucose oxidation in muscle [47,48]. Transgenic mice that overexpress malonyl-CoA decarboxylase in the liver have improved insulin sensitivity in skeletal muscle and this is primarily correlated to lowered levels of β-OHB-carnitine in muscle, whereas the muscle LC-CoA content was unchanged or even increased [49]. In accordance, knock out of peroxisome proliferator-activated receptor α, the transcriptional regulator of ketogenesis, protects against diet-induced insulin resistance in obese mice [50]. During fasting ketosis, β-OHB-carnitine in muscle is derived mainly from the ketone body β-OHB [51] and is likely to reflect the intracellular level of β-OHB. These findings could imply that high levels of ketones is causally linked to the development of insulin resistance, or alternatively that it is a metabolic marker of mitochondrial dysfunction [49]. In our study, we found very high levels of β-OHB in the fasting muscle and a concomitant increase in β-OHB-carnitine, demonstrating that ketone bodies were metabolized in skeletal muscle. Thus, it is possible that ketone bodies play a role in the development of insulin resistance during prolonged fasting by substrate competition. However, insulin resistance in obese participants during a 12 h fast does not appear to involve oxidation of ketone bodies, but can instead be caused by impairment in insulin signaling or other unknown factors.

There were no changes in NAD+, NADH, NADP+, FAD and AMP, ADP, ATP associated with prolonged fasting. Thus, myocellular energetics were preserved and there were no suggestions of deficient substrate oxidation and obvious mitochondrial defects despite insulin resistance. This is in agreement with previous measurements of mitochondrial function [52] and phosphorylation of AMP-activated protein kinase (AMPK) in lean participants during prolonged fasting [4,19]. In obese participants, the levels of the TCA-cycle intermediates citrate, succinate, and malate were reduced compared to lean independently of the duration of the fast, and this could indicate a reduction in the capacity for substrate oxidation. However, acetylcarnitine would accumulate if acetyl-CoA formation, either as end product of glycolysis or β-oxidation, exceeds its entry into the tricarboxylic (TCA) cycle, and this was not observed. In contrast, acetylcarnitine in obese were lower during 12 h of fasting, but normalized to levels similar to lean during 72 h of fasting. We do therefore not have evidence to suggest reduced mitochondrial function in either obese or lean participants.

Endogenous glucose production (EGP), consisting of hepatic glycogenolysis and both hepatic and renal gluconeogenesis were reduced during prolonged fasting. This has previously been observed in lean participants [4,53] and is most likely due to depletion of liver glycogen without a compensatory increase in gluconeogenesis [54]. In agreement with previous reports [6], glucose production in the non-insulin-stimulated state was lower in obese than lean during 12 h of fasting. This may reflect the higher plasma insulin levels in obese participants. In both obesity and fasting, insulin suppressed EGP indicating that, in contrast to muscle, hepatic sensitivity to insulin was preserved. Hepatic glycogen content is higher in obese than lean individuals [55], but it is completely depleted within the first two days of fasting [56]. In agreement with this, we found reduced EGP in both lean and obese participants during prolonged fasting in the non-insulin-stimulated state.

The capacity for glycogenesis in the obese, measured as insulin-stimulated NOGD during 12 h fast, was reduced by ~45%, but this did not translate into lower glycogen content. During prolonged fasting, glycogen levels in skeletal muscle were reduced ~20% in both groups. These findings confirm previous observations [57,58], but contrast our previous findings of minor increases in glycogen content in skeletal muscle from lean healthy individuals during 72 h of fasting [4]. Unlike our previous investigation, the skeletal muscle used for this experiment were freeze-dried prior to glycogen measurements. It is therefore likely that the discrepancy relies on fasting effects on water content in the muscle biopsies. Glycogen synthesis is regulated by GS and this enzyme is inactivated by phosphorylation. Similar to lean participants [4], GS phosphorylation increased during prolonged fasting also in obese. Insulin stimulation during 72 h of fasting decreased glucose oxidation and GS phosphorylation indicating that glucose incorporation into glycogen was prioritized. In lean, this may also be associated with lower glycogen breakdown because maltotriose, a metabolite of glycogenolysis [32], was reduced during prolonged fasting in lean only to levels comparable to obese. This suggests that the metabolic adaptation to prolonged fasting involving changes in glycogen breakdown are lost in muscle from insulin resistant obese participants.

The study has limitations. We did not monitor the participant’s lifestyle prior to the study. We have to rely on the self-reported information, but in these the participants declared to be weight stable with no changes in their life style (diet, level of exercise) three months prior to the study. For the muscle biopsies, we used a non-targeted metabolomics approach as opposed to targeted metabolomics. It is therefore not possible to determine the absolute content of the metabolites in the biopsies, and the single biopsy does not allow us to determine fluxes of substrates.

During the HEC, insulin levels were ~15–30% higher in obese than lean, which is a limitation for interpreting the reduction in EGP upon insulin stimulation, different responses in NOGD, AKT and AS160 signalling during HEC. The relatively high rate of ketogenesis and gluconeogenesis may exceed oxidation of the glucose and ketone bodies produced and can have led to a slight overestimation of lipid oxidation during prolonged fasting.

We have expressed metabolic fluxes determined by tracer techniques and indirect calorimetry per LBM, however adipose tissue is a metabolically active tissue and therefore fluxes may be overestimated in obese participants. To allow the readers to make their own interpretation of these data, we have included the glucose tracer data presented per TBW.

### Conclusion

In conclusion, the metabolic signatures in human skeletal muscle during acute and chronic insulin resistance reveal distinct differences. Impaired insulin signaling in skeletal muscle may subserve insulin resistance in obesity, whereas insulin resistance associated with prolonged fasting is more likely caused by substrate competition. Thus, the pathogenesis of insulin resistance in human participants is context specific, which most likely also determines its clinical significance.

## Supporting information

S1 Table Supplementary myocellular metabolomics. Metabolite concentrations were determined in skeletal muscle tissue during non-insulin-stimulated conditions in lean and obese after 12 h (control condition) and 72 h of fasting (fasted condition). The data are presented as fold of change. The muscle biopsies were obtained at t = 60 min. Green indicates significant difference (*p*≤0.05) between the mean values of the groups compared; metabolite ratio of < 1.00. Light green indicates narrowly missed statistical cutoff for significance 0.05<p<0.10; metabolite ratio of < 1.00. Red indicates significant difference (*p*≤0.05) between the groups compared; metabolite ratio of ≥ 1.00. Light Red indicates narrowly missed statistical cutoff for significance 0.05<p<0.10: metabolite ratio of ≥ 1.00. Non-colored cell indicates that mean values are not significantly different for that comparison. Blue indicates significant (*p*≤0.05) ANOVA effect.
(DOCX)

S1 File Supplementary methods section. Supplementary methods section regarding the metabolomics data.
(DOCX)

## Abbreviations

ACC: acetyl CoA Carboxylase
ALAT: alanine-aminotransferase
AMPK: AMP-activated protein kinase
AS160: Akt substrate of 160 kDa
BMI: body mass index
DAG: diacylglycero
EE: resting energy expenditure
EGP: endogenous glucose production
FFA: free fatty acids
G6P: glucose-6-phosphate
GS: glycogen Synthase
GIR: glucose infusion rate
HEC: hyperinsulinemic euglycemic clamp
LBM: lean body mass
NOGD: non-oxidative glucose disposal
R_a_: rate of appearance
R_d_: rate of disappearance
RER: respiratory exchange ratio
TG: triacylglyceride
