# Supplementary material for: Prolonged fasting-induced metabolic signatures in human skeletal muscle of lean and obese men
Source: PLoS One. 2018 Sep 5;13(9):e0200817. doi: 10.1371/journal.pone.0200817 (PMC6124727; doi:10.1371/journal.pone.0200817)
Supplement: S1 Table — Metabolite concentrations were determined in skeletal muscle tissue during non-insulin-stimulated conditions in lean and obese after 12 h (control condition) and 72 h of fasting (fasted condition). The data are presented as fold of change. The muscle biopsies were obtained at t = 60 min. Green indicates significant difference (p≤0.05) between the mean values of the groups compared; metabolite ratio of < 1.00. Light green indicates narrowly missed statistical cutoff for significance 0.05<p<0.10; metabolite ratio of < 1.00. Red indicates significant difference (p≤0.05) between the groups compared; metabolite ratio of ≥ 1.00. Light Red indicates narrowly missed statistical cutoff for significance 0.05<p<0.10: metabolite ratio of ≥ 1.00. Non-colored cell indicates that mean values are not significantly different for that comparison. Blue indicates significant (p≤0.05) ANOVA effect. (DOCX) [file pone.0200817.s001.docx]

**Table S1. Supplemental myocellular metabolomics**

| **Super Pathway** |  |  | **Fold of Change**  **ANOVA Contrasts** | | | | **Two-Way ANOVA w/**  **Repeated Measures** | | |
| --- | --- | --- | --- | --- | --- | --- | --- | --- | --- |
|  | **Sub Pathway** | **Biochemical Name** | **Fasted Control** | | **Obese Lean** | | **Fasting**  **Main**  **Effect** | **BMI**  **Main**  **Effect** | **Fasting**  **BMI**  **Inter-action** |
|  |  |  | **Lean** | **Obese** | **Control** | **Fasted** |  |  |  |
|  |  | 1,5-anhydroglucitol (1,5-AG) | 0.97 | 1.08 | 0.86 | 0.97 |  |  |  |
|  |  | glucose 1-phosphate | 1.28 | 1.00 | 0.67 | **0.52** |  |  |  |
|  | Glycolysis, Gluconeogenesis, and Pyruvate Metabolism | Isobar: fructose 1,6-diphosphate, glucose 1,6-diphosphate, myo-inositol 1,4 or 1,3-diphosphate | 0.95 | 1.71 | 0.67 | 1.21 |  |  |  |
|  |  | 3-phosphoglycerate | 0.89 | **1.41** | **0.65** | 1.03 |  |  |  |
|  |  | phosphoenolpyruvate (PEP) | 0.84 | 1.65 | 0.5 | 0.99 |  |  |  |
|  |  | glycerate | 0.91 | 1.13 | 0.84 | 1.03 |  |  |  |
| Carbohydrate | Pentose Metabolism | xylonate | 0.99 | 0.94 | 1.06 | 1.01 |  |  |  |
|  |  | xylose | **0.73** | 1.03 | 1.02 | **1.43** |  |  |  |
|  |  | arabitol | **0.46** | **0.57** | **0.68** | 0.85 |  |  |  |
|  |  | fructose | 0.8 | 0.88 | 0.82 | 0.91 |  |  |  |
|  | Fructose, Mannose | sorbitol | 1.01 | 0.83 | 1.13 | 0.93 |  |  |  |
|  | and | mannose | 1.01 | 1.11 | 1.07 | 1.18 |  |  |  |
|  | Galactose Metabolism | mannose-6-phosphate | 1.23 | 1.06 | 0.72 | **0.62** |  |  |  |
|  |  | mannitol | 0.92 | 0.97 | 0.93 | 0.98 |  |  |  |
|  |  | galactitol (dulcitol) | 0.96 | 1.14 | 0.65 | 0.78 |  |  |  |
|  | Nucleotide Sugar | guanosine 5'-diphospho-fucose | 1.1 | 1.19 | 0.8 | 0.86 |  |  |  |
|  | Aminosugar Metabolism | erythronate* | 1.05 | 1.16 | 1.13 | 1.24 |  |  |  |
| Energy | TCA Cycle | succinylcarnitine | 0.88 | 0.94 | 1.02 | 1.08 |  |  |  |
|  |  | fumarate | 1.08 | 1.19 | 0.87 | 0.95 |  |  |  |
| Lipid | Medium Chain Fatty Acid | caproate (6:0) | 0.84 | 1.2 | 0.8 | 1.15 |  |  |  |
|  |  | heptanoate (7:0) | 0.97 | 1.15 | 0.85 | 1,00 |  |  |  |
|  |  | caprylate (8:0) | 1.02 | 1.11 | 0.82 | 0.89 |  |  |  |
|  |  | pelargonate (9:0) | 0.88 | 1.21 | 0.76 | 1.04 |  |  |  |
|  |  | caprate (10:0) | **0.78** | 0.96 | 0.93 | 1.14 |  |  |  |
|  |  | laurate (12:0) | 0.89 | 0.92 | 0.93 | 0.95 |  |  |  |
|  | Long Chain Fatty Acid | myristate (14:0) | 0.99 | 0.98 | 0.98 | 0.97 |  |  |  |
|  |  | myristoleate (14:1n5) | 1.31 | **1.65** | 0.99 | 1.25 |  |  |  |
|  |  | pentadecanoate (15:0) | 0.98 | **1.19** | 0.92 | 1.12 |  |  |  |
|  |  | palmitoleate (16:1n7) | **1.66** | **1.97** | 1.02 | 1.21 |  |  |  |
|  |  | margarate (17:0) | 1.1 | 1.14 | 0.93 | 0.96 |  |  |  |
|  |  | 10-heptadecenoate (17:1n7) | 1.23 | **1.59** | 0.94 | 1.22 |  |  |  |
|  |  | cis-vaccenate (18:1n7) | 1,00 | 1.26 | 0.86 | 1.08 |  |  |  |
|  |  | 10-nonadecenoate (19:1n9) | **1.87** | **2.07** | 0.94 | 1.04 |  |  |  |
|  |  | eicosenoate (20:1n9 or 11) | **1.71** | **2.26** | 0.65 | 0.86 |  |  |  |
|  |  | erucate (22:1n9) | 0.95 | **1.6** | **0.49** | 0.82 |  |  |  |
|  |  | stearidonate (18:4n3) | 0.8 | 0.83 | 0.88 | 0.9 |  |  |  |
|  |  | eicosapentaenoate (EPA; 20:5n3) | 0.82 | 1.52 | 0.62 | 1.15 |  |  |  |
|  |  | docosapentaenoate (n3 DPA; 22:5n3) | 1,00 | **1.76** | 0.84 | 1.48 |  |  |  |
|  |  | docosahexaenoate (DHA; 22:6n3) | 0.99 | **1.56** | 0.57 | 0.89 |  |  |  |
|  |  | linoleate (18:2n6) | 1.16 | **1.65** | 0.76 | 1.08 |  |  |  |
|  | Polyunsaturated Fatty | linolenate [alpha or gamma; (18:3n3 or 6)] | 1.19 | **1.4** | 0.88 | 1.04 |  |  |  |
|  | Acid (n3 and n6) | dihomo-linolenate (20:3n3 or n6) | 1.08 | **1.66** | 0.84 | 1.29 |  |  |  |
|  |  | arachidonate (20:4n6) | 0.86 | **1.8** | 0.77 | 1.6 |  |  |  |
|  |  | adrenate (22:4n6) | **0.62** | 0.97 | 0.98 | **1.53** |  |  |  |
|  |  | docosapentaenoate (n6 DPA; 22:5n6) | 0.75 | 1.89 | 0.61 | 1.53 |  |  |  |
|  |  | docosadienoate (22:2n6) | **1.33** | **2.06** | 0.66 | 1.02 |  |  |  |
|  |  | dihomo-linoleate (20:2n6) | **1.57** | **1.83** | 0.91 | 1.06 |  |  |  |
|  |  | mead acid (20:3n9) | 1.3 | **1.65** | 1.21 | 1.52 |  |  |  |
|  | Fatty Acid, Branched | 13-methylmyristic acid | 1.06 | 1.15 | 0.88 | 0.95 |  |  |  |
|  |  | 15-methylpalmitate (isobar with 2-methylpalmitate) | 1.08 | **1.17** | 0.93 | 1.01 |  |  |  |
|  | Fatty Acid, Dicarboxylate | 2-hydroxyglutarate | 1.02 | 1.7 | 0.62 | 1.03 |  |  |  |
|  |  | azelate (nonanedioate) | 0.85 | 0.87 | 0.99 | 1.01 |  |  |  |
|  |  | undecanedioate | 0.99 | 1,00 | 1.14 | 1.14 |  |  |  |
|  |  | tetradecanedioate | **2.34** | 1.17 | 1.16 | **0.58** |  |  |  |
|  |  | hexadecanedioate | **1.92** | **1.35** | 1.01 | **0.71** |  |  |  |
|  |  | octadecanedioate | **1.45** | 1.12 | 1.04 | 0.8 |  |  |  |
|  |  | 3-carboxy-4-methyl-5-propyl-2-furanpropanoate (CMPF) | 1.26 | 1.25 | 0.46 | 0.45 |  |  |  |
|  | Fatty Acid, Amide | palmitic amide | 0.86 | 1.43 | 0.58 | 0.96 |  |  |  |
|  | Fatty Acid, Amino | 2-aminooctanoate | 1.08 | 0.73 | 0.72 | 0.49 |  |  |  |
|  | Fatty Alcohol, Long Chain | 1-hexadecanol | 0.59 | 0.76 | 0.66 | 0.85 |  |  |  |
|  | Fatty Acid Synthesis | malonylcarnitine | 1.05 | **1.3** | **0.78** | 0.97 |  |  |  |
|  | Fatty Acid Metabolism (also BCAA Metabolism) | butyrylcarnitine | 0.69 | 1.16 | 0.71 | 1.21 |  |  |  |
|  |  | propionylcarnitine | 1.22 | 1.42 | 0.92 | 1.07 |  |  |  |
|  |  | valerylcarnitine | **0.54** | 0.86 | 0.85 | 1.35 |  |  |  |
|  | Fatty Acid Metabolism | hexanoylcarnitine | 0.56 | 0.74 | 0.91 | 1.2 |  |  |  |
|  | (Acyl Carnitine) | octanoylcarnitine | 0.62 | 0.66 | 1.05 | 1.11 |  |  |  |
|  |  | decanoylcarnitine | 0.68 | 0.67 | 1.09 | 1.08 |  |  |  |
|  |  | laurylcarnitine | 0.67 | 0.57 | 1.41 | 1.21 |  |  |  |
|  |  | myristoylcarnitine | 0.81 | 0.54 | 1.73 | 1.16 |  |  |  |
|  |  | stearoylcarnitine | 0.64 | 0.65 | 1.21 | 1.21 |  |  |  |
|  |  | oleoylcarnitine | 0.89 | 0.76 | 1.49 | 1.27 |  |  |  |
|  | Carnitine Metabolism | deoxycarnitine | 0.91 | 0.99 | 0.86 | 0.93 |  |  |  |
|  |  | 3-dehydrocarnitine* | 0.96 | 0.98 | 0.92 | 0.94 |  |  |  |
|  | Fatty Acid, Monohydroxy | 2-hydroxypalmitate | 1.05 | **1.36** | 0.79 | 1.03 |  |  |  |
|  |  | 2-hydroxystearate | 1.06 | 1.42 | **0.7** | 0.94 |  |  |  |
|  |  | 13-HODE + 9-HODE | 1.17 | 1.01 | 1.13 | 0.98 |  |  |  |
|  | Inositol Metabolism | myo-inositol | 0.89 | 1.08 | 1.05 | **1.27** |  |  |  |
|  |  | chiro-inositol | 1.15 | 1.11 | 1.3 | 1.26 |  |  |  |
|  |  | scyllo-inositol | 0.92 | 1.08 | 1.1 | 1.29 |  |  |  |
|  |  | inositol 1-phosphate (I1P) | 1.08 | **1.3** | 1.03 | 1.24 |  |  |  |
|  | Phospholipid Metabolism | choline | **1.15** | **1.38** | 1.01 | 1.22 |  |  |  |
|  |  | choline phosphate | 1.08 | **1.24** | 0.97 | 1.1 |  |  |  |
|  |  | glycerophosphorylcholine (GPC) | **0.79** | 1.16 | **1.43** | **2.12** |  |  |  |
|  |  | ethanolamine | 0.87 | 0.95 | 0.9 | 0.99 |  |  |  |
|  |  | phosphoethanolamine | 0.98 | **1.63** | 0.75 | 1.25 |  |  |  |
|  | Lysolipid | 1-myristoyl- glycerophosphocholine (14:0) | **0.76** | 0.89 | 0.83 | 0.97 |  |  |  |
|  |  | 2-myristoyl- glycerophosphocholine* | 0.83 | **0.79** | **1.26** | 1.19 |  |  |  |
|  |  | 1-palmitoyl- glycerophosphocholine (16:0) | 0.85 | 1.05 | 0.9 | 1.11 |  |  |  |
|  |  | 2-palmitoyl- glycerophosphocholine* | **0.74** | 0.92 | 0.98 | 1.24 |  |  |  |
|  |  | 1-palmitoleoyl- glycerophosphocholine (16:1)* | 0.88 | 1.09 | 0.95 | 1.18 |  |  |  |
|  |  | 2-palmitoleoyl- glycerophosphocholine* | 0.92 | 1.03 | 1.29 | **1.46** |  |  |  |
|  |  | 1-stearoyl- glycerophosphocholine (18:0) | 0.74 | 0.76 | 0.75 | 0.77 |  |  |  |
|  |  | 2-stearoyl- glycerophosphocholine* | **0.56** | 0.91 | **0.76** | 1.23 |  |  |  |
|  |  | 1-oleoyl- glycerophosphocholine (18:1) | 0.92 | 1.05 | 0.96 | 1.09 |  |  |  |
|  |  | 2-oleoylglycerophosphocholine* | 1.04 | 1.11 | 1.29 | 1.38 |  |  |  |
|  |  | 1-linoleoyl- glycerophosphocholine (18:2n6) | **0.75** | 1.05 | 0.76 | 1.07 |  |  |  |
|  |  | 2-linoleoyl- glycerophosphocholine* | **0.86** | 0.96 | 1.06 | **1.19** |  |  |  |
|  |  | 1-linolenoyl- glycerophosphocholine (18:3n3)* | **0.73** | 0.85 | 0.94 | 1.09 |  |  |  |
|  |  | 2-linolenoyl-glycerophosphocholine(18:3n3)* | 0.9 | 0.9 | 1.44 | **1.44** |  |  |  |
|  |  | 1-eicosatrienoyl- glycerophosphocholine (20:3)* | 0.83 | 0.93 | 0.99 | 1.1 |  |  |  |
|  |  | 2-eicosatrienoyl- glycerophosphocholine* | 0.96 | 1,00 | 0.84 | 0.88 |  |  |  |
|  |  | 1-arachidonoyl- glycerophosphocholine (20:4n6)* | 0.76 | 0.89 | 0.9 | 1.06 |  |  |  |
|  |  | 2-arachidonoyl- glycerophosphocholine* | 1.12 | 0.99 | 0.97 | 0.86 |  |  |  |
|  |  | 1-docosapentaenoyl- glycerophosphocholine (22:5n3)* | 0.74 | 0.93 | 0.9 | 1.13 |  |  |  |
|  |  | 2-docosapentaenoyl- glycerophosphocholine (22:5n3)* | 0.99 | 0.9 | 1.11 | 1.01 |  |  |  |
|  |  | 2-docosahexaenoyl- glycerophosphocholine* | 0.97 | 0.73 | 0.71 | 0.54 |  |  |  |
|  |  | 1-palmitoyl- plasmenylethanolamine* | **0.61** | **1.77** | 0.69 | **1.99** |  |  |  |
|  |  | 1-oleoylplasmenylethanolamine* | 0.72 | **1.8** | 0.76 | **1.88** |  |  |  |
|  |  | 1-palmitoyl- glycerophosphoethanolamine | **0.55** | 1.31 | **0.6** | 1.44 |  |  |  |
|  |  | 1-stearoyl- glycerophosphoethanolamine | 0.66 | **1.89** | 0.66 | **1.9** |  |  |  |
|  |  | 1-oleoyl- glycerophosphoethanolamine | 0.73 | **1.53** | **0.68** | 1.42 |  |  |  |
|  |  | 2-oleoyl- glycerophosphoethanolamine* | 1.02 | 1.13 | 1.21 | 1.35 |  |  |  |
|  |  | 1-linoleoylglycero- phosphoethanolamine* | 0.82 | 1.64 | 0.83 | **1.66** |  |  |  |
|  |  | 2-linoleoyl- glycerophosphoethanolamine* | 0.93 | **1.64** | 0.92 | **1.61** |  |  |  |
|  |  | 2-linolenoylglycero- phosphoethanolamine (18:3n3)* | **0.67** | 1.08 | 1.31 | **2.12** |  |  |  |
|  |  | 1-arachidonoyl- glycerophosphoethanolamine* | 0.92 | 1.43 | 0.77 | 1.19 |  |  |  |
|  |  | 2-arachidonoyl- glycerophosphoethanolamine* | 1.04 | 0.95 | 1,00 | 0.91 |  |  |  |
|  |  | 2-docosapentaenoyl- glycerophosphoethanolamine* | 0.92 | 1,00 | 1.11 | 1.2 |  |  |  |
|  |  | 2-docosahexaenoyl- glycerophosphoethanolamine* | 0.96 | 0.97 | 0.73 | 0.74 |  |  |  |
|  |  | 1-eicosatrienoyl- glycerophosphoethanolamine* | 0.75 | 1.49 | 0.75 | 1.5 |  |  |  |
|  |  | 1-eicosapentaenoyl- glycerophosphoethanolamine* | 0.77 | **1.29** | 0.66 | 1.12 |  |  |  |
|  |  | 2-eicosapentaenoyl- glycerophosphoethanolamine* | 0.97 | 0.96 | 0.9 | 0.9 |  |  |  |
|  |  | 1-docosahexaenoyl- glycerophosphoethanolamine* | 0.74 | 1.22 | **0.49** | 0.82 |  |  |  |
|  |  | 1-palmitoyl- glycerophosphoinositol* | 0.98 | 1.22 | 0.85 | 1.05 |  |  |  |
|  |  | 1-stearoylglycerophosphoinositol | 0.9 | 1.65 | 0.84 | 1.54 |  |  |  |
|  |  | 1-oleoylglycerophosphoinositol* | 1.16 | **1.78** | 0.76 | 1.17 |  |  |  |
|  |  | 1-arachidonoyl- glycerophosphoinositol* | 1.01 | **1.61** | 0.89 | 1.42 |  |  |  |
|  |  | 1-stearoylglycerophosphoserine* | 0.75 | 1.26 | 0.81 | 1.35 |  |  |  |
|  |  | 1-arachidonoylglyercophosphate | 0.82 | 1.02 | 0.9 | 1.12 |  |  |  |
|  |  | 1-palmitoylglycerophospho- glycerol* | 1.27 | 1.05 | 1.18 | 0.98 |  |  |  |
|  |  | 1-stearoylglycerophospho- glycerol | **0.78** | 1.06 | 0.88 | 1.19 |  |  |  |
|  |  | 1-oleoylglycerophosphoglycerol* | **1.29** | **1.36** | 1.03 | 1.08 |  |  |  |
|  |  | 2-oleoylglycerophosphoglycerol* | **1.52** | **1.4** | 1.15 | 1.06 |  |  |  |
|  | Glycerolipid | glycerol 3-phosphate (G3P) | **0.8** | 1.15 | 1.03 | **1.49** |  |  |  |
|  | Monoacylglycerol | 1-palmitoylglycerol (1-monopalmitin) | 1.25 | **1.53** | 0.9 | 1.09 |  |  |  |
|  |  | 2-palmitoylglycerol (2-monopalmitin) | 0.98 | 1.27 | 0.84 | 1.09 |  |  |  |
|  |  | 1-stearoylglycerol  (1-monostearin) | 0.97 | 1.23 | 0.9 | 1.15 |  |  |  |
|  |  | 1-oleoylglycerol (1-monoolein) | **1.41** | **1.75** | 0.94 | 1.17 |  |  |  |
|  |  | 1-linoleoylglycerol (1-monolinolein) | 0.86 | **1.61** | **0.61** | 1.14 |  |  |  |
|  |  | 2-linoleoylglycerol (2-monolinolein) | 1.07 | 1.19 | 0.85 | 0.95 |  |  |  |
|  |  | 1-linolenoylglycerol | 1.21 | 1.19 | 1.16 | 1.14 |  |  |  |
|  |  | 1-arachidonylglycerol | 1.04 | 1.12 | 0.85 | 0.92 |  |  |  |
|  |  | 2-arachidonoyl glycerol | 0.82 | 1.13 | **0.72** | 0.99 |  |  |  |
|  |  | 1-docosahexaenoylglycerol (1-monodocosahexaenoin) | 0.78 | 1.11 | **0.43** | 0.61 |  |  |  |
|  | Sphingolipid Metabolism | sphinganine | 0.87 | **1.39** | 0.79 | 1.25 |  |  |  |
|  |  | palmitoyl sphingomyelin | 0.98 | 1.05 | 0.87 | 0.94 |  |  |  |
|  |  | stearoyl sphingomyelin | 0.9 | 1.12 | 0.92 | 1.15 |  |  |  |
|  |  | sphingosine | 1.06 | 1.15 | 0.94 | 1.02 |  |  |  |
| Nucleotide |  | inosine 5'-monophosphate (IMP) | 1.26 | 1.34 | 1.11 | 1.18 |  |  |  |
|  |  | inosine | 1.07 | 1.02 | 1.14 | 1.09 |  |  |  |
|  | Purine Metabolism, | hypoxanthine | 0.95 | 1.12 | 0.99 | 1.16 |  |  |  |
|  | (Hypo)Xanthine/Inosine | xanthine | **2.17** | 0.96 | 1.56 | 0.69 |  |  |  |
|  | containing | urate | **1.51** | **1.55** | 0.98 | 1.01 |  |  |  |
|  |  | allantoin | **0.33** | **0.48** | 0.93 | 1.32 |  |  |  |
|  | Purine Metabolism, | adenosine 3',5'-cyclic monophosphate (cAMP) | 0.92 | 1.13 | 0.85 | 1.05 |  |  |  |
|  | Adenine containing | adenosine | 0.81 | 0.92 | 0.91 | 1.04 |  |  |  |
|  |  | adenine | 0.99 | 1.01 | 0.97 | 1,00 |  |  |  |
|  |  | N1-methyladenosine | 0.9 | 0.97 | 0.92 | 0.99 |  |  |  |
|  | Purine Metabolism, | guanosine 5'- monophosphate (5'-GMP) | 1.03 | 0.85 | 1.04 | 0.85 |  |  |  |
|  | Guanine containing | guanosine-2',3'-cyclic monophosphate | **1.45** | **1.75** | 0.98 | 1.19 |  |  |  |
|  |  | guanosine | 1.18 | 1,00 | 1.32 | 1.12 |  |  |  |
|  |  | uridine | 1.11 | 1.07 | 1.08 | 1.04 |  |  |  |
|  | Pyrimidine Metabolism, | pseudouridine | 0.87 | 1.01 | 0.88 | 1.03 |  |  |  |
|  | Uracil containing | 5,6-dihydrouracil | 0.99 | 1.31 | 1.05 | **1.39** |  |  |  |
|  |  | beta-alanine | 1.16 | 0.78 | **1.6** | 1.07 |  |  |  |
|  | Pyrimidine Metabolism, | cytidine 5'-monophosphate (5'-CMP) | **1.18** | **1.28** | 0.97 | 1.06 |  |  |  |
|  | Cytidine containing | cytidine | 1.25 | 1.27 | 1.08 | 1.1 |  |  |  |
|  | Pyrimidine Metabolism, Thymine containing | 3-aminoisobutyrate | **1.25** | **1.69** | 0.75 | 1.01 |  |  |  |
|  | Purine and Pyrimidine Metabolism | methylphosphate | 0.95 | 1.1 | 0.98 | 1.13 |  |  |  |
|  | Nicotinate and | nicotinamide | 0.93 | 0.99 | 0.91 | 0.98 |  |  |  |
| Cofactors and | Nicotinamide Metabolism | adenosine 5'diphosphoribose | 0.86 | 0.86 | 1.04 | 1.04 |  |  |  |
| Vitamins | Pantothenate and CoA Metabolism | pantothenate | **0.86** | 1.06 | **0.77** | 0.95 |  |  |  |
|  |  | coenzyme A | 0.92 | 1.1 | 0.87 | 1.05 |  |  |  |

Metabolite concentrations were determined in skeletal muscle tissue during non-insulin-stimulated conditions in lean and obese after 12 h (control condition) and 72 h of fasting (fasted condition). The data are presented as fold of change. The muscle biopsies were obtained at t = 60 min. Green indicates significant difference (*p*≤0.05) between the mean values of the groups compared; metabolite ratio of < 1.00. Light green indicates narrowly missed statistical cutoff for significance 0.05<p<0.10; metabolite ratio of < 1.00. Red indicates significant difference (*p*≤0.05) between the groups compared; metabolite ratio of ≥ 1.00. Light Red indicates narrowly missed statistical cutoff for significance 0.05<p<0.10: metabolite ratio of ≥ 1.00. Non-colored cell indicates that mean values are not significantly different for that comparison. Blue indicates significant (*p*≤0.05) ANOVA effect.
