# Supplementary material for: Prolonged fasting-induced metabolic signatures in human skeletal muscle of lean and obese men
Source: PLoS One. 2018 Sep 5;13(9):e0200817. doi: 10.1371/journal.pone.0200817 (PMC6124727; doi:10.1371/journal.pone.0200817)
Supplement: S1 File — Supplementary methods section regarding the metabolomics data. (DOCX) [file pone.0200817.s002.docx]

In this study metabolomic profiling was performed by Metabolon Inc. (Durham, NC, USA) as previously described (1). Samples were analyzed using an untargeted gas chromatography–mass spectrometry and liquid chromatography–mass spectrometry based metabolomic quantification protocol.

Metabolon received 36 freeze dried human non-insulin stimulated muscle biopsy tissue samples, 9 from lean participants after a 12 h fast, 9 from obese participants after a 12 h fast, 9 from lean participants after a 72 h fast and 9 from obese participants after a 72 h fast.

Following receipt, samples were inventoried and immediately stored at -80^o^C. At the time of analysis, samples were prepared for analysis using Metabolon’s ISE (Intact Sample Extraction) solvent extraction method. The extracted samples were split into equal parts for analysis on the GC/MS and LC/MS/MS platforms. Also included were several technical replicate samples created from a homogeneous pool containing a small amount of all study samples.

**Instrument and Process Variability**

| ***QC Sample*** | ***Measurement*** | ***Median RSD*** |
| --- | --- | --- |
| Internal Standards | Instrument Variability | 5 % |
| Endogenous Biochemicals | Total Process Variability | 13 % |

Instrument variability was determined by calculating the median relative standard deviation (RSD) for the internal standards that were added to each sample prior to injection into the mass spectrometers. Overall process variability was determined by calculating the median RSD for all endogenous metabolites (i.e., non-instrument standards) present in 100% of the Client Matrix samples, which are technical replicates of pooled client samples. Values for instrument and process variability meet Metabolon’s acceptance criteria as shown in the table above.

Following normalization to client provided tissue weights, median scaling, imputation of missing values, if any, with the minimum observed value for each compound and log transformation median scaled data, ANOVA contrasts were used to identify biochemicals that differed significantly between experimental groups. Analysis by two-way ANOVA with repeated measures identified biochemicals exhibiting significant interaction and main effects for experimental parameters of treatment as 72 hour fasting and BMI category.

An estimate of the false discovery rate (*q*-value) was calculated to take into account the multiple comparisons that normally occur in metabolomic-based studies.
